# Interaction of Dietary Linoleic Acid and α-Linolenic Acids with rs174547 in *FADS1* Gene on Metabolic Syndrome Components among Vegetarians

**DOI:** 10.3390/nu11071686

**Published:** 2019-07-23

**Authors:** Yuan Kei Ching, Yit Siew Chin, Mahenderan Appukutty, Vasudevan Ramanchadran, Choo Yee Yu, Geik Yong Ang, Wan Ying Gan, Yoke Mun Chan, Lay Kek Teh, Mohd Zaki Salleh

**Affiliations:** 1 Department of Nutrition and Dietetics, Faculty of Medicine and Health Sciences, Universiti Putra Malaysia, Serdang 43400, Malaysia; 2 Research Centre of Excellence, Nutrition and Non-Communicable Diseases Universiti Putra Malaysia, Serdang 43400, Malaysia; 3 Programme of Sports Science, Faculty of Sports Science and Recreation, Universiti Teknologi MARA, Shah Alam 40450, Malaysia; 4 Malaysia Research Institute on Ageing (MyAgeing), Universiti Putra Malaysia, Serdang 43400, Malaysia; 5 Integrative Pharmacogenomics Institute (iPROMISE), Universiti Teknologi MARA, Puncak Alam 42300, Malaysia

**Keywords:** fatty acid desaturase 1 gene, single nucleotide polymorphism, vegetarians, metabolic syndromes, linoleic acid, *α*-linolenic acid

## Abstract

Fatty acid desaturase 1 (*FADS1*) gene controls the fatty acid metabolism pathway in the human body. The lower intake of α-linolenic acid (ALA) than linoleic acid (LA) among vegetarians may disrupt the fatty acid metabolism and limit the conversion of ALA to anti-inflammatory products such as eicosapentaenoic acid (EPA) and docosahexaenoic acid (DHA). This cross-sectional study aimed to determine the interaction of rs174547 in *FADS1* gene with LA and ALA on metabolic syndrome (MetS) components. A total of 200 Chinese and Indian vegetarians in Kuala Lumpur and Selangor, Malaysia participated in the present study. The data on socio-demographic characteristics, vegetarianism practices, dietary practices, anthropometric measurements, blood pressure (BP), and overnight venous fasting blood samples were collected from the vegetarians. The rs174547 in *FADS1* gene was significantly associated with MetS and its components such as waist circumference (WC) and fasting blood glucose (FBG). Multiple logistic regression analyses revealed that vegetarians with TT genotype of rs174547 in *FADS1* gene had higher odds for MetS, larger WC, higher BP, and a lower level of HDL-c. Two-way ANOVA analysis showed that LA interacts with rs174547 in *FADS1* gene to affect HDL-c (*p* < 0.05) among vegetarians. The present findings suggest the need to develop dietary guidelines for vegetarians in Malaysia. Prospective studies are also needed to affirm the interaction between LA and rs174547 in *FADS1* gene on HDL-c among Malaysian vegetarians.

## 1. Introduction 

Metabolic syndrome (MetS) is a cluster of risk factors that is associated with the development of cardiovascular disease (CVD) and type 2 diabetes mellitus (T2DM) [1]. To date, several MetS criteria had been introduced by different organizations from the years 1998 to 2009 [2,3,4,5]. The Joint Interim Statement (JIS) is the latest MetS definition that was introduced in the year 2009 [1]. Based on the JIS, MetS is defined by the presence of at least three out of the five MetS components, namely large waist circumference (WC), high blood pressure (BP), high levels of fasting blood glucose (FBG), high levels of triglyceride (TG), and low levels of high density lipoprotein cholesterol (HDL-c) [1]. The prevalence of MetS has been reported in many countries among the general population [6,7]. Several studies have reported that vegetarians are associated with a lower risk of MetS as compared to non-vegetarians [8,9,10]. Vegetarianism is a type of belief or eating practice, which depends on plant-based foods and abstaining from any form of animal foods [11]. Vegetarians are defined as individuals who practice vegetarianism, which can be further categorized into lacto-ovo vegetarians, ovo-vegetarians, lacto-vegetarians, and vegans based on their dietary intakes [12,13]. The protective effects of a vegetarian diet towards MetS may be associated with the high fibre, low macronutrients and low cholesterol intakes in vegetarians as compared to non-vegetarians [13,14,15]. While most studies reported a lower risk of MetS among vegetarians than non-vegetarians, MetS still occurs among the vegetarian population [8,9,10]. It was suggested that long duration of practicing a vegetarian diet may modify the fatty acid desaturase gene (*FADS*). In other words, *FADS* gene of these population who have adhered to a strict plant-based diet for many generations would have changed, and put them at increased risk of heart disease and colon cancer [16]. 

In comparison to those who practice non-vegetarian diet, the consumption of *α*-linolenic acid (ALA) among vegetarians is relatively low, as compared to linoleic acid (LA) [17]. ALA and LA are two precursors of essential fatty acids, that needs to be obtained from our daily diet, which can alter the enzyme activities of delta-5 desaturase (D5D) and delta-6 desaturase (D6D) [18,19]. D5D and D6D are two important rate limiting enzymes in the endogenous synthesis of long chain polyunsaturated fatty acids (LC-PUFAs) [20]. The endogenous synthesis of LC-PUFAs is controlled by fatty acid desaturase 1 (*FADS1*) and fatty acid desaturase 2 (*FADS2*) genes in the fatty acid metabolism pathway [20]. The ingested LA is then converted to γ-linolenic acid (GLA), dihomo-γ-linolenic acid (DGLA), and arachidonic acid (ARA); while ALA is converted to stearidonic acid (SDA), eicosatetraenoic acid (ETA), eicosapentaenoic acid (EPA), and docosahexaenoic acid (DHA) [20]. Notably, the converted ARA is a pro-inflammatory agent that promotes the development of atherosclerosis [21]. Conversely, the EPA and DHA are anti-inflammatory agents that inhibit the development of atherosclerosis [21]. Despite EPA and DHA being able to be converted from ALA, it should be noted that the endogenous synthesis of EPA and DHA from ALA is relatively slow and inefficient [17]. Only 5% to 10% of ALA is converted to EPA, and less than 2% to 5% of ALA is converted to DHA [17]. Furthermore, a systematic review had depicted the effectiveness of DHA and EPA in lowering the risk of MetS among the general population [22]. While fish is the main dietary sources of DHA and EPA, but fish is excluded from a vegetarian diet [21]. Therefore, DHA and EPA would be lacking in a vegetarian diet, and may increase the risk of MetS among vegetarians [23]. 

Over the past two decades, several genomic-wide association studies (GWAS) have reported the association of rs174547 in *FADS1* gene with MetS components as well as its interaction with LA and ALA on MetS components among the general population [19,24,25,26]. The rs174547 is a single nucleotide polymorphism (SNP) of the *FADS1* gene [20]. Based on the findings from a previous study, LA interacts with rs174547 in *FADS1* gene, which affects WC and HDL-c level among the general population [19]. Further, ALA interacts with rs174547 in *FADS1* gene to modify the level of HDL-c among the general population [25]. These results imply the importance of gene-diet interaction in the development of MetS components. However, the existing studies were conducted among the general population and focused on the associations of rs174547 in *FADS1* gene with WC, HDL-c and TG [19,24]. Little is known about the associations of rs174547 in *FADS1* gene with other MetS components, such as BP and FBG. Considering that BP and FBG are associated with LC-PUFAs level in the bloodstream [27,28,29], the interaction of rs174547 in *FADS1* gene with LA and ALA intakes may also be associated with BP and FBG levels. Furthermore, the dietary patterns of respondents from these existing studies were not reported [19,24,25,26], whereby it is possible that the respondents may have consisted of both vegetarians and non-vegetarians. Therefore, the extent of the influences of gene-diet interaction on MetS components, specifically in the vegetarian population, remains unknown. In addition, vegetarians have a different LC-PUFAs profile than non-vegetarians. Non-vegetarians who consume meat have significantly higher LC-PUFAs intakes, but lower n-6:n-3 intake ratios than vegetarians [30]. Thus, the findings from the general population may not be applicable to vegetarians due to the different LC-PUFAs intakes between vegetarians and non-vegetarians. Based on the study gaps, this cross-sectional study aimed to determine the association of rs174547 in *FADS1* gene with MetS and its components as well as the interaction between LA and ALA intakes and rs174547 in *FADS1* gene on MetS components among Malaysian vegetarians. 

## 2. Materials and Methods 

### 2.1. Study Design and Study Population

This cross-sectional study was approved by the Ethics Committee for Research involving Human Subjects, Universiti Putra Malaysia (JKEUPM) [Reference number: FPSK (FR16) P023]. The present study focused on Chinese and Indians vegetarians as vegetarianism is commonly practiced among Buddhist and Hindus followers in Malaysia [13,31]. Hence, a total of nine out of 31 religious related community centers in Kuala Lumpur and Selangor were randomly selected, based on the list of Chinese (Buddhist) and Indian (Hindu) religious related community centers that was obtained from the headquarters. The selected religious related community centers were the common places for Malaysian vegetarians to participate in their religious activities, particularly Buddhism and Hinduism. Members of the religious related community centers shared similar beliefs (Buddhism and Hinduism), and they are encouraged to adopt a vegetarian diet and abstain from the usage of alcohol and tobacco due to their religious belief. All members of the selected religious related community centers who fulfilled the study criteria, namely adults aged more than 18 years old, not pregnant or lactating, practicing vegetarianism for more than 2 years, not taking medications in controlling dyslipidaemia, diabetes, and hypertension, and not taking fatty acids supplements were invited to participate in the study. Written informed consents were obtained from respondents prior to data collection, and all respondents were requested to fast overnight in preparation for blood withdrawal on the morning of the data collection day. A total of 355 respondents consented to participate in the study. Of 355 consented respondents, 273 of them fulfilled the study criteria. The response rate of the present study was 76.9%. Among the 273 respondents, 232 of them completed the three-day dietary recall. However, 32 out of 232 respondents had misreported their energy intake based on the reported energy intake (EIrep):basal metabolic rate (BMR) ratio (EI_rep:_ BMR) [32]. Respondents with incomplete three-day dietary recall data and misreporting of energy intake were excluded from the present study. Hence, the final data of 200 respondents, who have completed the three-day dietary recall with accurate energy intake report, are presented in the present study.

### 2.2. Socio-Demographic Characteristic, Vegetarianism Practices and Dietary Intake Assessment

The socio-demographic characteristics (age, sex, and ethnicity), and information on vegetarianism practices (years of practicing vegetarianism, vegetarian categories and reasons for adopting vegetarianism) were collected using a self-administered questionnaire. Respondents were categorized into four vegetarian categories based on their current vegetarian practices [13], namely vegans (consume plant-based foods and no meat, fish, poultry, dairy products, and eggs); lacto-vegetarians (consume dairy products but no eggs, fish, meat, and poultry); ovo-vegetarians (consume eggs but no dairy products, fish, meat, and poultry), and lacto-ovo-vegetarians (consume dairy products and eggs, but no fish, meat, and poultry). 

Dietary intake of the respondents was assessed using a three-day dietary recall, which is comprised of two weekdays and one day on the weekend. The time of food consumption, a full description and quantity of food were interviewed by the researcher. The portion sizes of the foods consumed were estimated using household measurements. The dietary data of the respondents were analyzed using the Nutritionist Pro Software Version 2.4.1 (First Data Bank INC., 2011). The LA and ALA intakes were analyzed according to the United States Department of Agriculture (USDA) National Nutrient Database for Standard Reference [33]. Food items that were not included in the USDA Food Database were substituted by the foods in the Malaysian Food Composition database [34] and Singapore Food Composition Guide [35]. Dietary adequacy of the respondents was evaluated through the comparison of energy and nutrient intakes that are stated in the Recommended Nutrient Intakes (RNIs) for Malaysians, with the following recommended intakes for macronutrients (carbohydrate: 50.0–60.0%, protein: 10.0–20.0%, and fat: 25.0–30.0%) [36]. 

### 2.3. Anthropometric Measurements, Blood Pressure Measurements and Biochemical Analyses

The International Society for the Advancement of Kinanthropometry (ISAK) method was used for the anthropometric measurements. Body weight and height of the respondents were measured using a SECA213 portable stadiometer (SECA, Hamburg, Germany) in centimeter (cm), and TANITA Digital Weight Scale HD306 (TANITA Corporation, Arlington Heights, IL, USA) in kilogram (kg). WC was measured using a Lufkin tape W606PM (Lufkin, Lufkin, TX, USA) in cm. BP was measured using an Omron automatic blood pressure monitor (HEM-7121, Omron Corporation, Kyoto, Japan) in millimeter of mercury (mmHg). A total of 10.0 ml overnight fasting venous blood sample was taken from the respondents by a registered nurse. The collected blood samples were tested using an Olympus Au analyzer (AU640, Beckman Olympus, Brea, CA, USA) for blood glucose and blood lipid profile (TG and HDL-c).

### 2.4. Selection of Single Nucleotide Polymorphism (SNP) and Genotyping 

**Figure 1:**
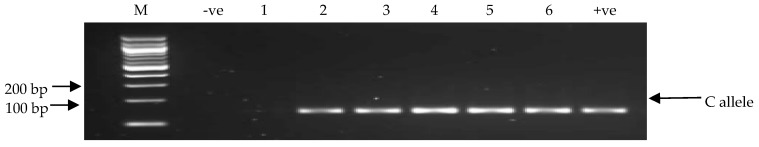
Representative genotyping data for C allele (144 bp). Note: M: 100 bp DNA Ladder; bp: base pair; -ve: negative control; +ve: positive control.

**Figure 2:**
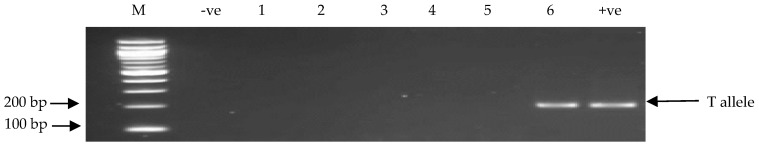
Representative genotyping data for T allele (191 bp). Note: M: 100 bp DNA Ladder; bp: base pair; -ve: negative control; +ve: positive control.

The rs174547 in *FADS1* gene was selected based on its associations and interaction with LC-PUFAs intake on MetS components [11,23,24], and its high linkage disequilibrium (LD) with other SNPs such as rs174546 and rs174537 [14,32] in the *FADS* gene cluster. Genomic DNA was extracted from the collected fasting venous blood using HiYield Genomic DNA Mini Kit (Real Biotech Corporation, Taiwan). The purity and quantity of the extracted genomic DNAs were checked using the NanoDrop 2000 spectrophotometer (Thermo Scientific, Waltham, MA, USA). To determine the C/T polymorphisms of rs174547 in *FADS1* gene, allele-specific polymerase chain reaction (PCR) was used to genotype the rs174547 in this study, with the following primers 5′-TTTGTTTTTGCTGTTTTCACCTACGAAT-3′ and 5′-AAGAACTCAGCGAACTCAGCACTCCACC-3′ for T allele, whilst the C allele was amplified using 5′-AAGGGACATGAGACTGTCTTGGTCAC-3′ and 5′-TGGAGCATAACACAACTATTGAAAATGG-3′ primers. The PCR reaction mix consisted of 0.5 µM of each primer, 1 X PCR buffer, 2 mM of MgCl_2_, 160 µM of the dNTPs mixture, 1 U of *Taq* DNA polymerase (Biotools, Madrid, Spain) and 50 ng of purified genomic DNA in a total volume of 20 µL. The amplification reaction was performed on T100 Thermal Cycler (Bio-Rad, Hercules, CA, USA) programmed with the following thermocycling conditions: Initial denaturation at 94 °C for 2 min followed by denaturation (94 °C for 20 s), annealing (63 °C for 20 s), and extension (72 °C for 30 s) for 30 cycles, and a final extension (72 °C for 5 min). The amplified PCR products were separated on a 2.5% agarose gel. The DNA fragments were stained with ethidium bromide and visualized using an ultraviolet (UV) with the G:Box system (Syngene, Saint-Quentin en Yvelines, France). Amplification of the T allele resulted in 191 bp PCR product, while the C allele produced 144 bp PCR product (Figure 1 and Figure 2).

### 2.5. Definition of MetS

Based on the Joint Interim Statement 2009 [1], respondents with at least three of the following MetS components, namely abdominal obesity with large WC (≥90.0 cm for men and ≥80.0 cm for women), high blood pressure (BP) [systolic blood pressure (SBP ≥130 mmHg), or diastolic blood pressure (DBP ≥85 mmHg), high triglyceride (TG) (≥1.7 mmol/L), high fasting blood glucose (FBG) ≥5.6 mmol/L), low levels of high density lipoprotein cholesterol (HDL-c) (<1.0 mmol/L for men and <1.3 mmol/L for women)] were considered as having MetS [1]. 

### 2.6. Statistical Analyses

Statistical analyses were performed using IBM SPSS Statistics 24.0 (SPSS Inc., Chicago, IL, USA) software. Continuous variables were presented as mean and standard deviation (means ± SD) for normally distributed data or median (interquartile range) for non-normally distributed data. Categorical variables were presented as frequency and percentage (n,%). Hardy-Weinberg equilibrium (HWE) was conducted by comparing the observed counts of different genotypes with their expected counts using a web-based tool, Court Lab Calculator [37]. Chi-square test was used to determine the associations of rs174547 in *FADS1* gene with MetS and its components. Multiple logistic regression analysis was used to determine the odds ratio of developing MetS and its components, after adjusting for age, sex, and ethnicity. The interaction of LA and ALA intake with rs174547 in *FADS1* gene on MetS components was tested using two-way ANOVA analysis. Log transformation was applied to obtain normally distributed data for FBG and TG when testing for interaction in two-way ANOVA analysis. The acceptance level of statistical significance for all tests was set at *p* < 0.05. 

## 3. Results 

General characteristics of the vegetarians are presented in Table 1. The present study was predominated by females (65.5%), Chinese (63.0%), and lacto-ovo-vegetarians (48.0%). The average age and years of practicing vegetarianism were 48.3 ± 13.2 years and 13.7 ± 9.2 years, respectively. The genotype frequencies for rs174547 in *FADS1* gene were CC (28.5%), CT (32.0%), and TT (39.5%), respectively. The distribution of rs174547 in *FADS1* gene was in accordance with the Hardy–Weinberg equilibrium (*p* > 0.05).

Table 2 shows the adequacy and nutrient intakes of vegetarians in the present study. The average energy intake among vegetarians was 7263 ± 1884 kJ/day. Based on the RNI 2017 [36], a majority of respondents (75.5%) did not meet the total RNI requirement. A total of 5.5% did not meet the recommended percentage of energy intake derived from carbohydrate and one in four vegetarians (25.5%) did not meet the protein recommendation. However, a higher proportion of fat intakes (43.5%) above recommendation (<30%) was observed in the present study among vegetarians. The mean carbohydrate, protein and fat intakes among vegetarians were 264.9 ± 74.7 g/day, 49.3 ± 15.1 g/day, and fat 57 ± 19 g/day, respectively. Meanwhile, the average intakes of SFA, MUFA, LA, and ALA were 26.0 ± 10.5 g/day, 25.3 ± 11.1 g/day, 18.2 ± 6.2 g/day, 7.6 (6.2–10.6) g/day, 7.9 ± 3.6 g/day, and 0.5 (0.4–0.8) g/day, respectively. 

Table 3 shows the distribution of vegetarians based on MetS and its components according to rs174547 in *FADS1* gene among vegetarians. The present study found that vegetarians with TT genotype of rs174547 in *FADS1* gene had larger WC, and lower level of HDL-c as well as a higher number of MetS components (*p* < 0.05). Besides, rs174547 in *FADS1* gene was significantly associated with WC, FBG, and MetS among vegetarians in the present study. 

Further analyses on the odds ratio of developing MetS and its components are presented in Table 4. Vegetarians with TT genotype of rs174547 in *FADS1* gene had higher odds for MetS (OR: 3.57, 95% CI: 1.02–12.47), larger WC (OR: 4.73, 95% CI: 1.41–15.93), higher BP (OR: 3.17, 95% CI: 1.05–9.61), and lower level of HDL-c (OR: 3.82, 95% CI: 1.22–11.98) compared to vegetarians with CC genotype of rs174547 in *FADS1* gene, after the multiple logistic regression models were adjusted for age, sex, and ethnicity.

Table 5 and Table 6 display the interactions of rs174547 in *FADS1* gene with LA and ALA intakes on MetS components. After classifying the vegetarians based on the tertile of LA intake [lowest (≤5.86 g/day), middle (5.87–8.19 g/day), and highest (≥8.20 g/day)], the present study found that vegetarians with the TT genotype of rs174547 in *FADS1* gene had larger WC in all LA intake groups while a higher FBG level and lower HDL-c level were found among vegetarians with middle LA intake group (*p* < 0.05). Meanwhile, a significant interaction was detected between rs174547 in *FADS1* gene and LA intake for HDL-c among vegetarians (*p* < 0.05). In contrast, there was no interaction of rs174547 in *FADS1* gene with dietary LA intake on other MetS components (Table 5). In terms of ALA, the present study found that vegetarians with the TT genotype of rs174547 in *FADS1* gene had larger WC in all ALA intake groups (*p* < 0.05), and a lower HDL-c level in middle ALA intake group, after classifying the vegetarians according to the tertile of ALA intake [lowest (≤0.45 g/day), middle (0.46–0.64 g/day), and highest (≥0.65 g/day)]. In addition, the present study found that there was no interaction of rs174547 in *FADS1* gene with ALA intakes on MetS components (*p* > 0.05) (Table 6).

## 4. Discussion 

To the best of our knowledge, this is the first study to report the association of rs174547 in *FADS1* gene with MetS among Malaysian vegetarians, which may serve as baseline data for future researchers. While there were several studies that had reported the nutrient intakes of Malaysian vegetarians [13,15,31], none of these studies had explored the LA and ALA intakes among Malaysian vegetarians. It is important to know the daily intake of LA and ALA due to the important roles of LA and ALA in the endogenous synthesis of LC-PUFAs. In recent years, several studies have replicated the association between rs174547 in *FADS1* gene and MetS components, as well as the interaction of rs174547 in *FADS1* gene with dietary LA and ALA intake on MetS components among the general population in various ethnicities [19,24,25,26], but there are no published data among the vegetarians. 

In the present study, TT genotype of rs174547 had the highest frequency (39.5%), followed by CT genotype (32.0%) and CC genotype (28.5%) in *FADS1* gene, which is consistent with previous studies [19,25], but contradicts with some studies conducted among the general population from other countries [24,26]. In terms of energy intake, the mean total energy intake of vegetarians was lower than previous studies conducted among Malaysian vegetarians [13,15]. The present study has pointed out that, total energy intake was insufficient among the vegetarians, whereby three in four vegetarians (75.5%) did not achieve the daily total RNI requirement. This result was in accordance with a previous local vegetarian study [15]. Despite Wong and colleagues’ [15] suggestion that lower reported energy intake could be related to under-reporting of the foods consumed by the vegetarians, it should be noted that in the present study, those with misreporting of total energy intake were excluded from the analysis. Therefore, the possibility of underreporting may be low. The present findings found that more than half of the vegetarians met the recommended intakes of carbohydrate (65.5%) and protein (74.5%) according to RNI for Malaysians [36], which are consistent with previous studies conducted among Malaysian vegetarians [13,15]. The sufficient intake of carbohydrate may likely be due to the high intake of starchy foods, such as rice, noodles, and root tubers among vegetarians [31], while the common protein sources for vegetarians are soya bean in various forms, such as soft curd, fried “tau kua”, and “fu chok” among Malaysian vegetarians [31].

Fat deficiency is rare among the Malaysian population. The main concern would be excessive fat intake due to high intakes of fried foods and processed foods in the local context [36,38]. The present study revealed that about two in five vegetarians (43.5%) exceeded the recommended percentage of energy intake derived from fat. A possible reason of excessive fat intake could be the high frequency intakes of meat analogues among Malaysian vegetarians. A previous Malaysian study reported that meat and seafood analogues were popular foods in vegetarians’ diet, as more than half of the vegetarians consumed these foods at least once a week [31]. While meat analogues can be part of a good source of plant protein, it should be noted that meat analogues are categorized as processed foods. Furthermore, “deep frying” is the most common cooking method for meat analogues, which may contribute to high fat intake among vegetarians. This implies that a well-planned vegetarian diet together with suitable cooking method are important in maintaining good health among vegetarians. 

In relation to LA and ALA, the present study found that percent of total energy (%) for LA (4.1%) and ALA (0.3%) met the recommended intakes for LA (3.0 to 7.0%) and ALA (0.3–1.2%) as suggested in RNI for Malaysian [36]. However, the daily intake of LA and ALA were lower than the dietary reference intake (DRI) for Americans [39]. Indeed, LC-PUFAs, including LA and ALA, play important roles in maintaining human health such as the development and functioning of the brain, heart, tissues, and organs of the human body [40]. Hence, it is imperative to raise awareness of consuming sufficient amount of LA and ALA among Malaysian vegetarians. A specific nutrition education programme on dietary LA and ALA intake is needed for vegetarians, in order to educate them on the importance of a balanced diet, as well as the health impacts of various nutrient intakes towards the human body. 

Based on the JIS 2009, the prevalence of MetS in Malaysian vegetarians was 21.0%, which is lower than the prevalence of MetS among the general population in Malaysia (42.5%) [41]. In terms of MetS components, the prevalences for each of the MetS components were lower than the general population as depicted in the National Health and Morbidity Survey 2015 (NHMS 2015) [42]. Meanwhile, the present study found that vegetarians with TT genotype of rs174547 in *FADS1* gene had a higher number of MetS components, and a higher prevalence of MetS than vegetarians with CC and CT genotypes in bivariate analysis. The logistic regression analysis also revealed that vegetarians with TT genotype of rs174547 in *FADS1* gene had higher odds (OR: 3.57 95% CI: 1.02-12.47) of developing MetS than vegetarians with CC genotype of rs174547 in *FADS1* gene after adjusting for age, sex, and ethnicity. These results indicated that TT genotype of rs174547 in *FADS1* gene could be a possible risk factor for MetS among Malaysian vegetarians. To date, there are no published data on the association between rs174547 in *FADS1* gene and MetS among vegetarians, which makes the direct comparison impossible. In terms of WC, about one in three vegetarians (35.5%) had large WC, with a higher prevalence of large WC found among vegetarians with TT genotype of rs174547 in *FADS1* gene. The present finding was in agreement with past studies that were conducted among the general population [19,24]. In addition, the logistic regression analysis in the present study found that vegetarians with TT genotype had higher odds (OR: 4.73, 95% CI: 1.41–15.93) of developing large WC than vegetarians with CC genotype, after adjusting for age, sex, and ethnicity. These findings suggest that the TT genotype of rs174547 in *FADS1* gene could be a risk genotype for large WC among vegetarians. 

The present study found that one in three vegetarians (30.0%) had low level of HDL-c. In the present study, vegetarians with TT genotype of rs174547 in *FADS1* gene had higher odds of developing lower HDL-c level compared to vegetarians with CC genotype of rs174547 in *FADS1* gene. This result warrants the needs to monitor dyslipidaemia status among vegetarians, especially among vegetarians with TT genotype of rs174547 in *FADS1* gene with a lower HDL-c level. The vegetarians, in this study, with TT genotype of rs174547 in *FADS1* gene had a lower level of HDL-c than their counterparts, which was consistent with a past study conducted among the China general population [43], but contradicts previous studies that revealed significant associations of CC genotype of rs174547 in *FADS1* gene with a lower level of HDL-c [26,43]. The effects of SNP on HDL-c might vary according to ethnic background. For instance, in the Mongolian population, the rs174547 in *FADS1* gene was not associated with HDL-c, while the CC of rs174547 in *FADS1* gene was associated with decreased HDL-c levels in the Japanese population [26]. Considering this is the first study that explored the association of rs174547 in *FADS1* gene with HDL-c among Chinese and Indian vegetarians in Malaysia, more studies are needed to affirm the results in the present study. Consistent with a past study that was conducted among patients with schizophrenia and bipolar disorder [44], the present study found that rs174547 in *FADS1* gene was associated with FBG in vegetarians. However, it is noted that mental health is recognised as one of the risk factors for MetS [45], therefore, it is possible that the association found between rs174547 in *FADS1* gene and FBG in the previous study could be confounded by the presence of depression [44]. Thus, more studies are needed to affirm the association of rs174547 in *FADS1* gene and FBG among the vegetarian population in future.

Compared to previous studies that had successfully discovered the association between rs174546 and BP among the general population [28], the present study found that the rs174547 in *FADS1* gene was not associated with BP, although rs174576 was in strong LD with rs174547 [19]. The insignificant association of rs174547 in *FADS1* gene with BP could be related to the small sample size of the present study. Besides, the insignificant association could also be due to the discrepancy in respondents’ characteristics. Notably, the past study that had successfully uncovered the association of genetic variation with BP and FBG was focused on young children whose age ranged from 2 to 10 years old [29]. However, the age of the respondents from this former study was much lower as compared to the average mean age of the vegetarians in the present study (48.3 years old). As age is one of the non-modifiable risk factors associated with BP [46], the discrepancy in age may confound the association between a genetic variant and BP. Considering limited studies on the association between rs174547 in *FADS1* gene and BP, more studies are required to determine the association between rs174547 in *FADS1* gene and BP among vegetarians.

The present study found that the dietary intake levels of LA may modify the association between rs174547 in *FADS1* gene and HDL-c among vegetarians, which is consistent with a past study that was conducted among the general population [19]. The interaction of dietary LA intake with rs174547 in *FADS1* gene on HDL-c may be explained by the decrease in *FADS1* activity among vegetarians with TT genotype of rs174547 and high LA intake, which may promote the accumulation of LA that subsequently inhibits the production of n-3 PUFAs. This may reduce the HDL-c raising effect among those individuals with TT genotype of rs174547 and high LA intake, and contribute to reduced HDL-c [19]. In other words, over-consumption of LA may inhibit the production of n-3 PUFAs, which reduces HDL-c level among those with TT genotype of rs174547 in *FADS1* gene [19]. While an interaction was observed between rs174547 in *FADS1* gene and LA on HDL-c, there were no interactions of dietary intake of LA with rs174547 in *FADS1* gene on other MetS components in the present study. In relation to ALA, the present study found that there was no interaction of dietary intake of ALA with rs174547 in *FADS1* gene on MetS components. These findings are in line with previous results [19], but contradicts with the study conducted by Hellstrand and colleagues [25]. This earlier study conducted among the Swedish population has shown that dietary intake of ALA modulates the association between rs174547 in *FADS1* gene and HDL-c, and the authors suggested that DHA, which comes from fatty fish, could be one of the possible factors that influences HDL-c [25]. Therefore, the unobservable interaction between ALA and rs174547 in *FADS1* gene on HDL-c may be attributed to the exclusion of fatty fish consumption from the vegetarians’ diet in the present study. Besides, the unobservable interactions of rs174547 in *FADS1* gene with LA and ALA may also be explained by the use of a three-day dietary recall as the dietary assessment tool. The short-term diet measurement by recall method may not reflect the usual intakes of LA and ALA of vegetarians. It is recommended that the serum fatty acid composition analysis, which reflects LC-PUFAs in the bloodstream, has a better credibility to reflect the quality and types of dietary fat, consumed over a long period of time [47]. 

The strength of this study includes detailed collection of actual foods and beverages consumption through three-day dietary recall by qualified nutritionist, whereby the common foods consumed by the Malaysian Chinese and Indians were able to be captured and analyzed. Nevertheless, there are several limitations that should be taken into consideration. Firstly, the present study was a cross-sectional study, which was not able to determine the causality effect between rs174547 in *FADS1* gene and MetS. Prospective studies are recommended to overcome this problem in the future. Besides, the present study focused on the *FADS1* gene and was restricted to the rs174547 in *FADS1* gene. However, the rs174547 in *FADS1* gene is strongly associated with the MetS components and had perfect LD with other SNPs. Therefore, the rs174547 acted as the ideal SNP to determine the gene-diet interaction on MetS components among Malaysian vegetarians. Next, the present study consisted of both Chinese and Indian vegetarians, which may affect the interaction of dietary intakes of LA and ALA with rs174547 on the MetS components due to the heterogeneity of the population. Future studies can focus on a single ethnicity and sex to avoid this heterogeneity issue. Lastly, the short-term diet measurement for three days (two weekdays and one day on weekend) may not be able to reflect the usual intakes of LA and ALA of the vegetarians. Future studies can use the serum fatty acid composition analysis to determine the LA and ALA in the human blood due to its better credibility in assessing the quality and types of fat consumed over an extended period. 

## 5. Conclusions

The rs174547 of *FADS1* gene was significantly associated with MetS and its components such as WC and FBG. Vegetarians with TT genotype of the rs174547in *FADS1* gene had higher odds of developing MetS, larger WC, higher BP, and lower level of HDL-c. Further investigations are needed in order to determine the possible linking mechanism between TT genotype of rs174547 in *FADS1* gene with MetS and its components. Meanwhile, the present study suggests that the dietary intake of LA may interact with rs174547 in *FADS1* gene to affect HDL-c level, which warrants the needs to monitor the amount of dietary LA intakes in vegetarians’ daily diet. In terms of policy, the present study suggests the need to develop dietary guidelines for vegetarians in Malaysia, and emphasized the balanced intakes of LA and ALA intake. Currently, several countries such as North America [48] and Japan [49] have developed specific dietary guidelines for vegetarians. Prospective studies such as cohort studies on gene-diet interaction are suggested in order to affirm the interaction between LA and rs174547 in *FADS1* gene on HDL-c among Malaysian vegetarians.

## Figures and Tables

**Table 1:** Socio-demographic, vegetarianism practices, and genotype distribution of rs174547 in *FADS1* gene of vegetarians (*n* = 200).

Variable	*n* (%)	Mean ± SD
**Age (years)**		48.3 ± 13.2
**Sex**		
Male	69 (34.5)	
Female	131 (65.5)	
**Ethnicity**		
Chinese	126 (63.0)	
Indians	74 (37.0)	
**Years of practising vegetarianism**		13.7 ± 9.2
**Vegetarianism categories**		
Vegans	38 (19.0)	
Lacto vegetarians	52 (26.0)	
Ovo vegetarians	14 (7.0)	
Lacto-ovo vegetarians	96 (48.0)	
**Genotype**		
CC	57 (28.50)	
CT	64 (32.0)	
TT	79 (39.5)	

**Table 2:** Energy, macronutrients, fibre and dietary fats intake of vegetarians (*n* = 200).

Variables	*n* (%)	Mean ± SD
^≠^ **Total energy intake (kJ)**		7263 ± 1884
% RNI	87.9 ± 21.1	
<RNI	151 (75.5)	
≥RNI	49 (24.5)	
^≠^ **Carbohydrate (g)**		264.9 ± 74.7
<50.0%	11 (5.5)	
50.0–65.0%	131 (65.5)	
>65.0%	58 (29.0)	
^≠^ **Protein (g)**		49.3 ± 15.1
<10.0%	51 (25.5)	
10.0–20.0%	149 (74.5)	
>20.0%	0 (0.0)	
^≠^ **Fat (g)**		57.1 ± 19.7
<25.0%	49 (24.5)	
25.0–30.0%	64 (32.0)	
>30.0%	87 (43.5)	
**Fibre (g)**		26.0 ± 10.5
**Cholesterol (mg)**		112.7 ± 119.6
**Saturated fat (g)**		25.3 ± 11.1
**MUFA (g)**		18.2 ± 6.2
^⏀^ **PUFA (g)**		7.6 (6.2–10.6)
**LA (g)**		7.9 ± 3.6
^⏀^ **ALA (g)**		0.5 (0.4–0.8)

Note: RNI: recommended nutrient intake; kJ: kilojoule; MUFA: monounsaturated fatty acid; PUFA: polyunsaturated fatty acid, LA: linoleic acid; ALA: *α*-linolenic acid. Variables are presented as Mean ± SD, and as *n* (%). ^≠^ Variables are presented in Mean ± SD. ^⏀^ Variables are presented in Median (IQR).

**Table 3:** Characteristics of the MetS and its components based on rs174547 in *FADS1* gene (*n* = 200).

Variable	CC (*n* = 57)	CT (*n* = 64)	TT (*n* = 79)	Total (n = 200)	*F*/$\chi^{2}$ Value	*p* Value
^≠^ **WC (cm)**						
Mean ± SD	76.6 ± 8.7	78.7 ± 10.8	84.8 ± 12.5	80.5 ± 11.5	11.01	0.0001 *
Normal WC	51 (89.5)	43 (67.2)	35 (44.3)	129 (64.5)	29.80	0.0001 *
Large WC	6 (10.5)	21 (32.8)	44 (55.7)	71 (35.5)		
^≠^ **SBP (mmHg)**						
Mean ± SD	126.4 ± 20.9	128.5 ± 17.0	127.8 ± 17.8	127.6 ± 18.4	0.21	0.811
Normal SBP	33 (57.9)	34 (53.1)	39 (49.4)	106 (53.0)	0.97	0.617
High SBP	24 (42.1)	30 (46.9)	40 (50.6)	94 (47.0)		
^≠^ **DBP (mmHg)**						
Mean ± SD	76.3 ± 11.9	76.3 ± 9.7	74.2 ± 10.8	75.5 ± 10.8	0.92	0.399
Normal DBP	42 (73.7)	52 (81.2)	64 (81.0)	158 (79.0)	1.36	0.507
High DBP	15 (26.3)	12 (18.8)	15 (19.0)	42 (21.0)		
**High BP**						
No	32 (56.1)	31 (48.4)	38 (48.1)	101 (50.5)	1.02	0.602
Yes	25 (43.9)	33 (51.6)	41 (51.9)	99 (49.5)		
^⏀^ **FBG (mmol/L)**						
Median (IQR)	4.7 (4.4–5.1)	4.7 (4.4–5.0)	4.9 (4.4–5.5)	4.7 (4.4–5.2)	3.14	0.082
Normal	49 (86.0)	59 (92.2)	61 (77.2)	169 (84.5)	6.18	0.045 *
High FBG	8 (14.0)	5 (7.8)	18 (22.8)	31 (15.5)		
^⏀^ **TG (mmol/L)**						
Median (IQR)	1.2 (0.8–1.8)	0.9 (0.7-1.5)	1.1 (0.7–1.8)	1.1 (0.8–1.7)	4.99	0.208
Normal TG	41 (71.9)	50 (78.1)	57 (72.2)	148 (74.0)	0.83	0.659
High TG	16 (28.1)	14 (21.9)	22 (27.8)	52 (26.0)		
^≠^**HDL-c** (mmol/L)						
Mean ± SD	1.3 ± 0.2	1.3 ± 0.3	1.2 ± 0.3	1.3 ± 0.3	3.76	0.025 *
Normal HDL-c	45 (78.9)	47 (73.4)	48 (60.8)	140 (70.0)	5.75	0.057
Low HDL-c	12 (21.1)	17 (26.6)	31 (39.2)	60 (30.0)		
^≠^ **MetS**						
Mean ± SD	1.2 ± 1.1	1.4 ± 1.0	2.0 ± 1.4	1.6 ± 1.2	8.29	0.0001 *
No	50 (87.7)	54 (84.4)	54 (68.4)	158 (79.0)	9.12	0.010 *
Yes	7 (12.3)	10 (15.6)	25 (31.6)	42 (21.0)		

Note: WC: waist circumference; SBP: systolic blood pressure; DBP: diastolic blood pressure; BP: blood pressure; FBG: fasting blood glucose; TG: triglyceride; HDL-c: high-density lipoprotein cholesterol; MetS: metabolic syndrome. The genotypes of rs174547 in FADS1 gene (CC, CT and TT) are independent variables, whilst MetS and its components (large waist circumference, high blood pressure, high fasting blood glucose, high triglyceride, and low level of high-density lipoprotein cholesterol) are dependent variables. ^≠^ Variables are presented as Mean ± SD, and tested by one-way ANOVA with value reported in *F* and *p*. ^⏀^ Variables are presented as median (IQR), and tested by Kruskal–Wallis test with value reported in $\chi^{2}$ and *p*. * Indicates a significant difference at *p* < 0.05.

**Table 4:** Multiple logistic regression analysis (*n* = 200).

Genotype	^a^ MetS	^b^ Large WC	^c^ High BP	^d^ High FBG	^e^ High TG	^f^ Low HDL-c
CC	1.00	1.00	1.00	1.00	1.00	1.00
CT	1.53 (0.52–4.50)	3.84 (1.37–10.71) *	2.04 (0.92–4.49)	0.63 (0.19–2.16)	0.71 (0.29–1.72)	1.39 (0.58–3.34)
TT	3.57 (1.02–12.47) *	4.73 (1.41–15.93) *	3.17 (1.05–9.61) *	2.04 (0.54–7.63)	0.78 (0.24–2.48)	3.82 (1.22–11.98) *

Note: WC: waist circumference; SBP: systolic blood pressure; DBP: diastolic blood pressure; BP: blood pressure; FBG: fasting blood glucose; TG: triglyceride; HDL-c: high-density lipoprotein cholesterol; MetS: metabolic syndrome. Six different multiple logistic regression models (a, b, c, d, e and f) were used to predict the odds ratio of developing MetS, large WC, high BP, high FBG, high TG and low level of HDL-c. All models of multiple logistic regression analysis adjusted odds ratios (95% confidence interval) are shown after adjustment for age, sex and ethnicity. * Indicates a significant difference at *p* < 0.05.

**Table 5:** Interaction between rs174547 in *FADS1* gene and LA intake on MetS components (*n* = 200).

Variables	LA (g/day) Low (≤5.86) (*n* = 67)			LA (g/day) Medium (5.87–8.19) (*n* = 65)			LA (g/day) High (≥8.20) (*n* = 68)			*p* Interaction
	Genotype	Mean ± SD	*p* Value	Genotype	Mean ± SD	*p* Value	Genotype	Mean ± SD	*p* Value	
WC (cm)	CC (*n* = 15)	78.9 ± 10.4	0.020 *	CC (*n* = 19)	77.3 ± 9.4	0.016 *	CC (*n* = 23)	74.5 ± 6.6	0.009 *	0.177
	CT (*n* = 23)	77.3 ± 11.4		CT (*n* = 22)	76.3 ±8.1		CT (*n* = 19)	83.1 ± 12.0		
	TT (*n* = 29)	85.3 ± 9.8		TT (*n* = 24)	85.3 ±15.1		TT (*n* = 26)	83.7 ±13.1		
SBP (mmHg)	CC (*n* = 15)	129.4 ± 19.4	0.467	CC (*n* = 19)	130.4 ± 23.5	0.722	CC (*n* = 23)	121.0 ± 19.3	0.611	0.535
	CT (*n* = 23)	135.4 ± 21.1		CT (*n* = 22)	126.0 ± 14.4		CT (*n* = 19)	123.2 ± 11.1		
	TT (*n* = 29)	128.5 ± 20.7		TT (*n* = 24)	129.6 ± 18.9		TT (*n* = 26)	125.3 ± 12.9		
DBP (mmHg)	CC (*n* = 15)	76.9 ± 11.9	0.350	CC (*n* = 19)	78.1 ± 13.4	0.811	CC (*n* = 23)	74.5 ± 10.8	0.904	0.882
	CT (*n* = 23)	77.9 ± 10.8		CT (*n* = 22)	76.2 ± 7.9		CT (*n* = 19)	74.5 ± 10.4		
	TT (*n* = 29)	73.4 ± 11.8		TT (*n* = 24)	76.0 ± 11.4		TT (*n* = 26)	73.4 ± 9.1		
Log FBG (mmol/L)	CC (*n* = 15)	0.7 ± 0.1	0.373	CC (*n* = 19)	0.7 ± 0.05	0.004 *	CC (*n* = 23)	0.7 ± 0.1	0.459	0.807
	CT (*n* = 23)	0.7 ± 0.04		CT (*n* = 22)	0.7 ± 0.04		CT (*n* = 19)	0.7 ± 0.03		
	TT (*n* = 29)	0.7 ± 0.1		TT (*n* = 24)	0.7 ± 0.1		TT (*n* = 26)	0.7 ± 0.1		
HDL-c (mmol/L)	CC (*n* = 15)	1.2 ± 0.2	0.213	CC (*n* = 19)	1.4 ± 0.3	0.001 *	CC (*n* = 23)	1.3 ± 0.2	0.205	0.005*
	CT (*n* = 23)	1.4 ± 0.3		CT (*n* = 22)	1.4 ± 0.3		CT (*n* = 19)	1.2 ± 0.2		
	TT (*n* = 29)	1.3 ± 0.3		TT (*n* = 24)	1.1 ± 0.3		TT (*n* = 26)	1.2 ± 0.3		
Log TG (mmol/L)	CC (*n* = 15)	0.2 ± 0.3	0.101	CC (*n* = 19)	0.01 ± 0.2	0036 *	CC (*n* = 23)	0.1 ± 0.3	0.460	0.075
	CT (*n* = 23)	0.1 ±0.2		CT *(n =* 22)	−0.1 ± 0.1		CT (*n* = 19)	0.1 ±0.3		
	TT (*n* = 29)	0.1 ± 0.2		TT (*n* = 24)	0.1 ± 0.3		TT (*n* = 26)	0.02 ± 0.3		

Note: WC: waist circumference; SBP: systolic blood pressure; DBP: diastolic blood pressure; FBG: fasting blood glucose; HDL-c: high-density lipoprotein cholesterol; TG: triglyceride; LA: linoleic acid. Data are presented as Mean ± SD, and tested by One-Way ANOVA analysis with value in *p*. Gene-diet interaction analysis tested by Two-Way AVOVA analysis with p-interaction term. * Indicates a significant difference at *p* < 0.05.

**Table 6:** Interaction between rs174547 in *FADS1* gene and ALA intake on MetS components (*n* = 200).

Variables	ALA Low (≤0.45) (*n* = 70)			ALA Medium (0.46–0.64) (*n* = 63)			ALA High (≥0.65) (*n* = 67)			*p* Interaction
	Genotype	Mean ± SD	*p* Value	Genotype	Mean ± SD	*p* Value	Genotype	Mean ± SD	*p* Value	
WC (cm)	CC (*n* = 21)	79.5 ± 10.7	0.035 *	CC (*n* = 16)	77.7 ± 5.8	0.009 *	CC (*n* = 20)	72.7 ± 7.2	0.004 *	0.258
	CT (*n* = 24)	76.5 ± 10.9		CT (*n* = 23)	79.2 ± 10.6		CT (*n* = 17)	80.9 ± 11.1		
	TT (*n* = 25)	84.8 ± 11.9		TT *(n =* 24)	86.0 ± 10.4		TT (*n* = 30)	83.7 ± 14.7		
SBP (mmHg)	CC (*n* = 21)	133.5 ± 20.8	0.531	CC (*n* = 16)	122.4 ± 16.6	0.391	CC (*n* = 20)	122.1 ± 22.9	0.558	0.434
	CT (*n* = 24)	130.4 ± 22.1		CT (*n* = 23)	128.6 ± 13.8		CT (*n* = 17)	125.8 ± 12.5		
	TT (*n* = 25)	126.6 ± 19.2		TT (*n* = 24)	129.3 ± 18.6		TT (*n* = 30)	127.6 ± 16.4		
DBP (mmHg)	CC (*n* = 21)	79.1 ± 13.5	0.264	CC (*n* = 16)	76.0 ± 9.3	0.272	CC (*n* = 20)	73.7 ± 11.9	0.981	0.413
	CT (*n* = 24)	75.0 ± 9.6		CT (*n* = 23)	79.5 ± 9.6		CT (*n* = 17)	73.9 ± 9.4		
	TT (*n* = 25)	73.8 ± 11.2		TT (*n* = 24)	74.6 ± 11.9		TT (*n* = 30)	74.2 ± 9.9		
Log FBG (mmol/L)	CC (*n* = 21)	0.7 ± 0.1	0.754	CC (*n* = 16)	0.7 ± 0.1	0.097	CC (*n* = 20)	0.7 ± 0.04	0.019 *	0.293
	CT (*n* = 24)	0.7 ± 0.03		CT (*n* = 23)	0.7 ± 0.04		CT (*n* = 17)	0.7 ± 0.04		
	TT (*n* = 25)	0.7 ± 0.1		TT (*n* = 24)	0.7 ± 0.1		TT (*n* = 30)	0.7 ± 0.1		
HDL-c (mmol/L)	CC (*n* = 21)	1.3 ± 0.2	0.200	CC (*n* = 16)	1.3 ± 0.3	0.043 *	CC (*n* = 20)	1.3 ± 0.2	0.254	0.230
	CT (*n* = 24)	1.4 ± 0.4		CT (*n* = 23)	1.3 ± 0.3		CT (*n* = 17)	1.2 ± 0.2		
	TT (*n* = 25)	1.3 ± 0.3		TT (*n* = 24)	1.2 ± 0.3		TT (*n* = 30)	1.2 ± 0.3		
Log TG (mmol/L)	CC (*n* = 21)	0.2 ± 0.3	0.212	CC (*n* = 16)	0.1 ± 0.2	0.226	CC (*n* = 20)	0.1 ± 0.2	0.981	0.410
	CT (*n* = 24)	0.04 ± 0.3		CT (*n* = 23)	−0.02 ± 0.2		CT (*n* = 17)	0.1 ± 0.2		
	TT (*n* = 25)	0.04 ± 0.2		TT (*n* = 24)	0.1 ± 0.3		TT (*n* = 30)	0.1 ± 0.3		

Note: WC: waist circumference; SBP: systolic blood pressure; DBP: diastolic blood pressure; FBG: fasting blood glucose; HDL-c: high-density lipoprotein cholesterol; TG: triglyceride; ALA: *α*-linolenic acid. Data are presented as Mean ± SD, and tested by One-Way ANOVA analysis with value in *p*. Gene-diet interaction analysis tested by Two-Way AVOVA analysis with p-interaction term. * Indicates a significant difference at *p* < 0.05.
